# Environmental and Climate Impacts on Physiological Aging: Comprehensive Pathways to Enhanced Age-related Resilience

**DOI:** 10.14336/AD.2024.0478

**Published:** 2025-06-27

**Authors:** Chengmeng Zhang, Shangjun Liu, Gong Chen

**Affiliations:** ^1^Institute of Population Research, Peking University, Beijing, China.; ^2^Oxford Institute of Population Ageing, Oxford, UK.; ^3^Centre for Longitudinal Studies, Social Research Institute, University College London, UK.; ^4^Institute of Ageing Studies, Peking University, Beijing, China.

**Keywords:** Aging, Organ-specific aging, Environmental impact, Climate change, Integrative health care

## Abstract

This perspective article highlights the pressing necessity to focus on heterogeneity in organ-specific aging, emphasizing the influence of environmental and climate changes, as well as social interactions, on the aging process of individual organs. It explores innovative and integrative strategies tailored to the unique aging mechanisms of different organs, aiming to preserve their biological clocks. By emphasizing the importance of addressing organ-specific vulnerabilities, the article calls for transcending traditional pharmacological treatments and organ transplants. It proposes a comprehensive approach that combines environmental management, social support systems, and advanced technological interventions to improve the well-being and longevity of aging populations.

## Introduction

Physiological aging of organs in murine models has been shown to trigger a functional decline across the body, potentially disrupting the healthy maintenance process and imposing additional burdens on healthcare systems [1]. Although these findings underscore critical biological mechanisms in specific species, they hold significant reference value for understanding human physiological aging. Recent advances in plasma proteomics, utilizing machine learning models to analyze blood plasma samples from 5,676 adults, have revealed that nearly 20% of individuals exhibit significantly accelerated aging in at least one organ, highlighting the variability in organ-specific aging processes [2]. This variability indicates that each organ ages according to its own unique pattern, affecting the overall health of individuals in distinct ways. This pivotal finding challenges the traditional understanding of aging as a uniform process across the body, and paves the way for healthcare solutions tailored to the unique requirements of individual organs [3].

The integration of genomics and systems biology has significantly advanced our understanding of aging, revealing how genetic predispositions, metabolic profiles, and environmental stressors influence physiological decline [1, 4]. Recent research has further elucidated the complex interactions between environmental stressors such as air pollution and green spaces and biomarkers involved in various organs. For instance, particulate matter and urban noise have been found to specifically alter biomarkers related to brain age and phenotypic age, underscoring the varied effects of environmental factors on different physiological systems [5]. These insights highlight the necessity of considering a broad range of environmental factors in aging studies, compelling the adoption of a multidisciplinary approach that integrates perspectives from biomedical engineering, computational modelling, and the social sciences. Such an approach is essential for crafting effective public health interventions aimed at mitigating the diverse impacts of environmental stressors on aging.

Understanding the distinct patterns of physiological aging across different organs reveals profound implications for both healthcare strategies and public health policies. Just as the human body follows a general health cycle, each organ operates on its own unique biological clock or 'organ health cycle,' which can vary significantly from one organ to another [6]. Recognizing these individual cycles is essential for developing targeted therapies that address specific aging processes effectively. By comprehending the specific aging pathways of various organs, health researchers can anticipate potential risks and intervene in precise therapies.

Moreover, these insights underscore the need to incorporate social and economic dimensions into healthcare solutions. Targeted interventions not only improve the quality of life for individuals, but also inform public health policies by highlighting disparities in environmental and social conditions. The implications of such findings extend beyond medical treatment into community-based preventive care. As our understanding of how specific organs respond to environmental stressors deepens, it becomes possible to develop targeted and integrated interventions that can mitigate these effects.

## Environmental Impacts on Physiological Aging

Human physiological aging and the resultant health status changes are intricately influenced by a combination of genetic components, environmental conditions, and random stochastic events [7]. Increasingly poor environmental conditions, such as air pollution, ultraviolet radiation, and climate change, play an important role in accelerating the aging processes of various organs, thereby posing significant health risks [8]. While some studies have laid a foundational understanding of differential aging progresses of various organs through lifestyle surveys [9-11], the underlying mechanisms remain complex and multifaceted. Nonetheless, the profound impact of exogenous environmental factors on the aging process is evident and warrants comprehensive investigation.

### Air Pollution and Physiological Aging of Different Organs

Air pollution is a key environmental factor that may exacerbates organ-specific physiological aging and increases the risk of chronic diseases associated with various organs. For instance, studies have shown that exposure to air pollution, especially particulate matter, can accelerate the aging of the lungs and increase the risk of chronic respiratory diseases [12, 13] . These fine particles (including carbon monoxide, sulfur dioxide, nitrogen dioxide, silica, asbestos and bioaerosols) induce inflammation and oxidative stress in lung tissues, contributing to premature lung aging and decreased pulmonary function. Additionally, air pollution has been linked to cardiovascular diseases, as fine particulate matter can enter the bloodstream, causing endothelial dysfunction and increasing the risk of atherosclerosis and other cardiovascular conditions [12]. Long-term exposure to polluted air may also negatively impacts the brain, potentially accelerating cognitive decline and increasing the risk of age related neurodegenerative diseases [14].

A study from the Global Burden of Disease (GBD) indicates that air pollution alone leads to over 6 million premature deaths annually, and 213 million disability-adjusted life years (DALYs) are attributed to exposure to air pollution each year [15]. Vulnerable populations, including older adults, children, and individuals with chronic respiratory conditions, face a significantly higher risk of health impacts related to air pollution [16, 17]. This underscores the importance of addressing air quality to mitigate its adverse effects on respiratory and cardiovascular health.

### UV Radiation and Physiological Aging

The health risks of ultraviolet (UV) radiation are an emerging topic of concern. While moderate exposure to UV radiation is generally harmless and even beneficial for processes like vitamin D synthesis, excessive UV exposure poses significant risks to various organs, particularly the skin and eyes [18]. Prolonged exposure to UV radiation has been linked to DNA damage in skin cells, resulting in photoaging and a higher incidence of skin cancers [19, 20]. High level of UV radiation is associated with a significantly increased incidence of skin cancers, highlighting the need for protective measures. UV radiation also degrades the skin’s protective barrier, making it more prone to environmental damage and cellular aging.

Excessive UV radiation impacts ocular health as well, making ocular cells more prone to senescence. A study on the use of UV-C germicidal lamps indicates that even brief exposure to UV light can cause irreversible harmful changes in skin cells and retinal cells in the eyes [21]. Moreover, chronic UV exposure can alter dopamine levels in the central nervous system and peripheral organs, including the skin, leading to cognitive deficits [22]. Recognizing the balance between beneficial and harmful effects of UV exposure is essential. While small amounts of UV light are beneficial, overexposure leads to significant health risks and accelerates the aging process of various organs.

### Climate Change Risks for Accelerated Organ-specific Aging

Climate change poses significant risks for accelerated physiological aging, particularly through extreme temperature fluctuations and heatwaves. In recent years, climate change and its alignment with Sustainable Development Goals (SDGs) have captured significant attention from both the academic community and the public [23]. Climate change, driven by human activities, poses considerable threats to human health, with older adults being especially susceptible to the dangers of heatwaves and other severe temperature fluctuations [24]. Moreover, climate change influences the aging process of organs across all age groups through long-term mechanisms, posing enduring health risks.

For instance, research highlights the negative effects of heat stress. In older adults, the cardiovascular response to heat stress is impaired, and their ability to regulate stroke volume during heat exposure is notably diminished [25]. While aging naturally diminishes the physiological functions of cells throughout the body, cells within the cardiovascular system, such as cardiomyocytes, are particularly susceptible to age-related deterioration [26]. This vulnerability may worsen due to various climate stressors, potentially hastening the decline of neurological and other organ-specific functions, highlighting the broader implications of rising global temperatures on human organ health. A mismatch between the rate of change in climate conditions and human beings' resilience to cope with these changes may severely affect overall health [27].

### Comprehensive Perspectives on Environmental Influences

A comprehensive understanding of environmental influences on physiological aging requires integrating both physical and social dimensions. Physical environmental factors like air pollution and UV radiation directly impact organ aging, while social factors and community support play significant roles in health outcomes. Beyond concerns about the heterogeneous aging of individual organs influenced by the physical environment, we must also consider the social attributes of physiological aging. Well-being, or happiness, often regarded as a central element of active aging, is thought to offer protection against the effects of body aging. This has been corroborated in biological experiments, for example, a study involving aging mice showed that those with active social lives experienced a delayed onset of age-related immune and oxidative stress compared to their isolated peers [28]. These findings underscore the potential of community-driven interventions to improve health and prolong life by creating supportive environments that mitigate the adverse effects of environmental stressors.

Furthermore, environmental inequalities result in varying health impacts across different populations. There is increasing recognition of the role of environmental inequalities in determining health outcomes. Not all populations experience these effects equally due to varying levels of pollution and access to green spaces, individuals in different regions, or even within the same city, might experience vastly different aging patterns. Although the relationship cannot be directly observed in the data, scholars believe residential greenspace or microclimates within communities play an important role in organ-specific health outcomes [10]. For instance, residents in communities with more green spaces exhibit healthier cardiopulmonary systems, as green spaces improve air quality and provide opportunities for physical activity [29]. Social connections and community involvement are vital in mitigating the adverse effects of environmental stressors, delaying age-related declines, and enhancing overall health [30]. These insights underscore the importance of fostering supportive environments that address both the physical and social aspects of aging, setting the stage for integrative community-based healthcare approaches.

These observations highlight a significant trend in considering environmental factors during health transitions, particularly given the heightened vulnerabilities faced by the aging population to air pollution. The academic community advocates the need to focus on biomarkers of accelerated organ-specific aging, such as increased epigenetic age, which highlights the growing sensitivity to environmental exposures among individuals [31]. Integrating knowledge from environmental science and public health into aging research allows us to address not only the biological determinants of aging but also the social and environmental factors that shape health outcomes. Based on the above discussion, we can develop interventions that mitigate the effects of aging across multiple organ systems and promote healthy aging holistically.

## Integrative Care Approaches: Community based Responses

To address the complex interplay between organ aging and environmental factors, we propose a paradigm shift towards integrative care. It is no longer sufficient to view organ transplantation, surgical interventions and prescribing medicines as the primary recourse for addressing the ramifications of aging [32, 33]. Previous research has indicated that factors such as natural and artificial light, noise, plants, art, air quality, color, simulated and actual views of nature, and support groups or other social opportunities could greatly benefit the healing process [34]. Therefore, we advocate for a dual strategy that marries environmental healing with medical care.

Environmental healing refers to the strategic implementation of public health and policy measures aimed at reducing exposure to harmful environmental conditions [35]. This encompasses initiatives such as mitigating air and water pollution, promoting urban green spaces, and enhancing physical factors considering changing climate patterns [36, 37]. For example, implementing stricter emissions standards for vehicles and industries can significantly reduce the burden of air pollution, while increasing the accessibility of green spaces in urban areas can provide a buffer against the urban heat island effect and promote physical activity. Parks, as a common environmental practice of green spaces, provide a range of natural benefits such as intercepting dust, toxins and noise, sequestering carbon and buffering flooding, creating place for recreation, fostering well-being, and a host of other social benefits [38].

An empirical study from Huanhuaxi Park in Chengdu, China, shows that different types of landscapes vary in their effectiveness at promoting physiological and psychological recovery. Topographical landscapes provided more restorative experiences, with water elements receiving the highest restoration evaluations [39]. Similarly, a case from Stanley Park, an urban forest park in Canada, demonstrates that therapeutic plant landscapes can engage visitors’ senses, alleviate physiological, emotional, and psychological stress, and offer comforting and enjoyable experiences [40]. These findings suggest that authorities should consider human health recovery needs in urban and rural community planning, and by strategically designing landscapes with therapeutic elements, these measures can improve the quality of life for residents.

Environment-oriented comprehensive care emphasizes a holistic approach to the health needs of the entire population [41]. It integrates preventive measures, lifestyle modifications, and personalized medical interventions to address the multifaceted aspects of aging. This approach may involve regular screening for organ-specific biomarkers, tailoring nutritional advice based on individual metabolic profiles, and leveraging digital health technologies for remote monitoring and early intervention.

Examples from countries such as New Zealand and the United Kingdom highlight the effectiveness of Green Prescriptions, also known as Nature Prescriptions, in integrating environmental elements with health promotion activities to enhance physical health and combat physiological aging [42]. Green Prescription involves doctors encouraging patients to engage in activities in natural settings to improve their mental and physical health, based on research indicating that regular contact with nature can reduce the dependency on certain medications [43, 44]. This reflects the therapeutic potential of nature-oriented integrative care in enhancing health.

Additionally, investing in adaptive infrastructure, such as green spaces, cool zones, and clean air shelters, can provide respite from environmental stressors and promote healthy aging. A recent study highlights the vulnerability of older adults with cognitive impairments, who may not be aware of the need to cool down, hydrate, or move to cooler areas during heatwaves, putting them at higher risk [45]. Given the importance of encouraging mobility and active lifestyles among older adults, it is also crucial to create accessible cool zones and green spaces within communities to support their well-being during extreme heat conditions.

From a macro policy perspective, initiatives such as the WHO Age-Friendly Cities Framework, the Active Ageing and AGE Platform Europe programmes advocate for the design of age-friendly spaces, promoting comprehensive development that fosters healthy aging environments. One study explores practical implementations, such as the Urban Therapy—Urban Health Path Project, which aims to develop criteria for selecting urban space features (e.g., topography, building elements, or small architecture) to determine their suitability for the rehabilitation of older adults. It also hypothesizes the development of technological tools to activate and engage older adults in physical activities and improve cognitive functions [46].

As we advocate for a paradigm shift towards an integrative care model, we emphasize blending environmental healing with comprehensive care to effectively address the challenges of aging related to demographic changes. This approach aims not only to reduce exposure to harmful environmental conditions but also to emphasize personalized medical interventions and lifestyle changes tailored to individual aging processes. Integrating environmental strategies with proactive health measures paves the way for creating sustainable, age-friendly environments that enhance the quality of life and functional capacity of older populations. The discussion will turn to identifying potential gaps in current strategies and proposing innovative approaches that ensure aging individuals receive the most effective care and support in constantly evolving environments.

## Envisioning the Future: Gaps and directions

Looking ahead, the future of aging research could benefit from harnessing the power of big data and artificial intelligence (AI) to develop predictive models of physiological aging. Establishing a robust surveillance system that monitors the health effects of environmental factors on older adults, with a particular focus on organ-specific biomarkers, is crucial [5]. By integrating multi-omics data with environmental exposure data and health records, we can identify the key drivers of differential organ aging and develop targeted interventions [47]. For instance, precision medicine approaches, which involve designing and refining diagnostics, treatments, and predictions using large, complex datasets that include individual genetics, functionalities, and environmental changes, have become mainstream [48]. Predictive models based on AI have been successfully utilized to forecast risks, employing deep learning algorithms to track multiple diseases, immune aging, frailty, and cardiovascular aging [49].

Harnessing the power of big data and AI, we can also integrate meteorological, environmental, and health data to develop early warning systems that predict the health impacts of climate change on older adults. Such systems would enable healthcare institutions and communities to take proactive measures to mitigate the negative consequences of climate change. Moreover, personalized risk assessments could be generated based on an individual's health profile, living conditions, and environmental exposures, empowering older adults to take preventive actions and make informed decisions about their health.

Another proposal is the creation of environmental healing tourism zones specifically designed for older adults. These areas would be situated in locations with optimal climatic conditions and minimal pollution, offering a therapeutic environment for organ recovery or self-healing. Planning for such zones involves collaborating with environmental experts to select sites that offer the best combination of natural resources and accessibility. Challenges such as land use regulations, sustainability of resources, and ensuring equitable access would need to be addressed through comprehensive management strategies that incorporate community input and scientific research. For instance, health-driven seasonal tourism has become a new mode of eldercare for older adults in China, with tourism services for older adults also being part of the silver economy policy framework of China [50]. In winter, many from the northern provinces of China prefer to spend their time in the warmer climates of the south (mainly Hainan) [51]. For these older adults, the health benefits of the destination become a significant attraction in tourism marketing. However, these destinations often face potential challenges in accommodating medical products or services. Thus, it is necessary to integrate scientific rehabilitation measures, such as hospitals, accessible environments, and physical landscape features, with the social attributes of the destination to create environmentally friendly, age-inclusive care communities that support the localized well-being of older adults. Such initiatives could combine eco-therapy, personalized nutrition, and mindfulness practices to holistically address the effects of climate change on organ aging. By providing older adults with opportunities for environmental healing, we can enhance their resilience and quality of life in the face of global climate change.

However, addressing the impact of climate change on organ-specific aging requires concerted efforts from individuals, communities, and policymakers, highlighting the need for interdisciplinary collaboration. Developing targeted educational campaigns and outreach programs is crucial to inform older adults about the health risks associated with environmental factors, especially climate change. These strategies could include creating informative digital content, organizing community workshops, and integrating climate health topics into existing public health initiatives, involving experts from public health, environmental science, urban planning, and technology fields. Additionally, it is essential to train healthcare professionals and community social workers, equipping them with the necessary skills and knowledge to address the diverse health implications of climate change. Such educational initiatives would empower a well-informed community to engage in preventive measures and adapt to the changing environment.

## Conclusion

In conclusion, the narrative of physiological aging in organs, shaped by both intrinsic biological processes and extrinsic environmental factors, demands a nuanced, multidisciplinary response. Integrating environmental healing into comprehensive care strategies offers a promising pathway not only for prolonging life but significantly enhancing the quality of life for older adults, particularly for organs susceptible to accelerated aging. Our discussions have highlighted the effective use of big data and AI in predicting and managing aging processes, suggesting these technologies as realistic and beneficial tools. This emphasizes the imperative of strengthening interdisciplinary collaborations among biomedical scientists, environmentalists, and policymakers.

Moreover, establishing environmental healing tourism zones and promoting targeted educational campaigns will further support community-based integrative care approaches. Embracing these strategies will not only mitigate the adverse effects of climate change on organ aging, but also empower communities to proactively support their aging populations. By addressing the impact of climate and environmental changes on organ aging preemptively, we can envision a future where healthy aging is not just an aspiration but a universal reality.

## Data Availability

As a perspective piece, this work does not address issues concerning the availability of data and materials.

## References

[1] Schaum N et al. (2020). Ageing hallmarks exhibit organ-specific temporal signatures. Nature, 583:596-602. 10.1038/s41586-020-2499-y PMC7757734 32669715

[2] Oh HS-H et al. (2023). Organ aging signatures in the plasma proteome track health and disease. Nature, 624:164-172. 10.1038/s41586-023-06802-1 PMC10700136 38057571

[3] Kozlov M (2023). Are your organs ageing well? The blood holds clues. Nature, 624:237-238. 10.1038/d41586-023-03821-w 38086942

[4] Calder PC, Carding SR, Christopher G, Kuh D, Langley-Evans SC, McNulty H (2018). A holistic approach to healthy ageing: how can people live longer, healthier lives? J Hum Nutr Diet, 31:439-450. 10.1111/jhn.12566 29862589

[5] Pu F et al. (2024). Heterogeneous associations of multiplexed environmental factors and multidimensional aging metrics. Nat Commun, 15:4921. 10.1038/s41467-024-49283-0 PMC11164970 38858361

[6] Prattichizzo F et al. (2024). Organ-specific biological clocks: Ageotyping for personalized anti-aging medicine. Ageing Res Rev, 96:102253. 10.1016/j.arr.2024.102253 38447609

[7] Gladyshev VN (2016). Aging: progressive decline in fitness due to the rising deleteriome adjusted by genetic, environmental, and stochastic processes. Aging Cell, 15:594-602. 10.1111/acel.12480 PMC4933668 27060562

[8] Brunet A (2020). Old and new models for the study of human ageing. Nat Rev Mol Cell Biol, 21:491-493. 10.1038/s41580-020-0266-4 PMC7531489 32572179

[9] Alugoju P, Krishna Swamy VKD, Anthikapalli NVA, Tencomnao T (2023). Health benefits of astaxanthin against age-related diseases of multiple organs: A comprehensive review. Crit Rev Food Sci Nutr, 63:10709-10774. 10.1080/10408398.2022.2084600 35708049

[10] Tian YE, Cropley V, Maier AB, Lautenschlager NT, Breakspear M, Zalesky A (2023). Heterogeneous aging across multiple organ systems and prediction of chronic disease and mortality. Nat Med, 29:1221-1231. 10.1038/s41591-023-02296-6 37024597

[11] Pataky MW, Young WF, Nair KS (2021). Hormonal and Metabolic Changes of Aging and the Influence of Lifestyle Modifications. Mayo Clin Proc, 96:788-814. 10.1016/j.mayocp.2020.07.033 PMC8020896 33673927

[12] Bernard SM, Samet JM, Grambsch A, Ebi KL, Romieu I (2001). The potential impacts of climate variability and change on air pollution-related health effects in the United States. Environ Health Perspect, 109:199-209. 10.1289/ehp.109-1240667 PMC1240667 11359687

[13] Eckhardt CM, Wu H (2021). Environmental Exposures and Lung Aging: Molecular Mechanisms and Implications for Improving Respiratory Health. Curr Environ Health Rep, 8:281-293. 10.1007/s40572-021-00328-2 PMC8567983 34735706

[14] Delgado-Saborit JM, Guercio V, Gowers AM, Shaddick G, Fox NC, Love S (2021). A critical review of the epidemiological evidence of effects of air pollution on dementia, cognitive function and cognitive decline in adult population. Sci Total Environ, 757:143734. 10.1016/j.scitotenv.2020.143734 33340865

[15] Murray CJL et al. (2020). Global burden of 87 risk factors in 204 countries and territories, 1990-2019: a systematic analysis for the Global Burden of Disease Study 2019. Lancet, 396:1223-1249. 10.1016/S0140-6736(20)30752-2 PMC7566194 33069327

[16] Kashtan Y et al. (2024). Nitrogen dioxide exposure, health outcomes, and associated demographic disparities due to gas and propane combustion by U.S. stoves. Sci Adv, 10:eadm8680. 10.1126/sciadv.adm8680 PMC11068006 38701214

[17] Vilcins D et al. (2024). Updates in Air Pollution: Current Research and Future Challenges. Ann Glob Health. 10.5334/aogh.4363 PMC10836163 38312715

[18] Yu S-L, Lee S-K (2017). Ultraviolet radiation: DNA damage, repair, and human disorders. Mol Cell Toxicol, 13:21-28.

[19] Salminen A, Kaarniranta K, Kauppinen A (2022). Photoaging: UV radiation-induced inflammation and immunosuppression accelerate the aging process in the skin. Inflamm Res, 71:817-831. 10.1007/s00011-022-01598-8 PMC9307547 35748903

[20] Panich U, Sittithumcharee G, Rathviboon N, Jirawatnotai S (2016). Ultraviolet Radiation-Induced Skin Aging: The Role of DNA Damage and Oxidative Stress in Epidermal Stem Cell Damage Mediated Skin Aging. Stem Cells Int, 2016:7370642. 10.1155/2016/7370642 PMC4842382 27148370

[21] Alessio N et al. (2024). Germicidal lamps using UV-C radiation may pose health safety issues: a biomolecular analysis of their effects on apoptosis and senescence. Aging (Albany NY), 16:7511-7522. 10.18632/aging.205787 PMC11131978 38700499

[22] Yoon K-N et al. (2024). Chronic ultraviolet irradiation induces memory deficits via dysregulation of the dopamine pathway. Exp Mol Med. 10.1038/s12276-024-01242-x PMC11263540 38825641

[23] Bain PG et al. (2019). Public views of the Sustainable Development Goals across countries. Nat Sustain, 2:819-825.

[24] Ebi KL et al. (2021). Extreme Weather and Climate Change: Population Health and Health System Implications. Annu Rev Public Health, 42:293-315. 10.1146/annurev-publhealth-012420-105026 PMC9013542 33406378

[25] Gravel H, Chaseling GK, Barry H, Debray A, Gagnon D (2021). Cardiovascular control during heat stress in older adults: time for an update. Am J Physiol Heart Circ Physiol, 320:H411-H416. 10.1152/ajpheart.00536.2020 33275528

[26] Abdellatif M, Rainer PP, Sedej S, Kroemer G (2023). Hallmarks of cardiovascular ageing. Nat Rev Cardiol, 20:754-777. 10.1038/s41569-023-00881-3 37193857

[27] Burraco P, Orizaola G, Monaghan P, Metcalfe NB (2020). Climate change and ageing in ectotherms. Glob Chang Biol, 26:5371-5381. 10.1111/gcb.15305 32835446

[28] Garrido A, Cruces J, Ceprián N, Corpas I, Tresguerres JA, De la Fuente M (2019). Social environment improves immune function and redox state in several organs from prematurely aging female mice and increases their lifespan. Biogerontology, 20:49-69. 10.1007/s10522-018-9774-4 30255225

[29] Yeager RA, Smith TR, Bhatnagar A (2020). Green environments and cardiovascular health. Trends Cardiovasc Med, 30:241-246. 10.1016/j.tcm.2019.06.005 PMC7995555 31248691

[30] White MP et al. (2021). Associations between green/blue spaces and mental health across 18 countries. Sci Rep, 11:8903. 10.1038/s41598-021-87675-0 PMC8076244 33903601

[31] Ward-Caviness CK et al. (2020). Accelerated epigenetic age as a biomarker of cardiovascular sensitivity to traffic-related air pollution. Aging (Albany NY), 12:24141-24155. 10.18632/aging.202341 PMC7762491 33289704

[32] Shah VH, Rao MK (2020). Changing Landscape of Solid Organ Transplantation for Older Adults: Trends and Post-Transplant Age-Related Outcomes. Curr Transplant Rep, 7:38-45.

[33] Drenth-van Maanen AC, Wilting I, Jansen PAF (2020). Prescribing medicines to older people—How to consider the impact of ageing on human organ and body functions. Br J Clin Pharmacol, 86:1921-1930. 10.1111/bcp.14094 PMC7495267 31425638

[34] Queen NJ, Hassan QN, Cao L (2020). Improvements to healthspan through environmental enrichment and lifestyle interventions: where are we now? Front Neurosci, 14:523670. 10.3389/fnins.2020.00605 PMC7325954 32655354

[35] DuBose J, MacAllister L, Hadi K, Sakallaris B (2018). Exploring the Concept of Healing Spaces. HERD, 11:43-56. 10.1177/1937586716680567 29514569

[36] Huisman ERCM, Morales E, van Hoof J, Kort HSM (2012). Healing environment: A review of the impact of physical environmental factors on users. Build Environ, 58:70-80.

[37] Zborowsky T (2014). The Legacy of Florence Nightingale's Environmental Theory: Nursing Research Focusing on the Impact of Healthcare Environments. HERD, 7:19-34. 10.1177/193758671400700404 25303425

[38] Dushkova D, Ignatieva M (2020). New trends in urban environmental health research: from geography of diseases to therapeutic landscapes and healing gardens. Geogr Environ Sustain, 13:159-171.

[39] Deng L et al. (2020). Empirical study of landscape types, landscape elements and landscape components of the urban park promoting physiological and psychological restoration. Urban For Urban Green, 48:126488.

[40] He M, Wang Y, Wang WJ, Xie Z (2022). Therapeutic plant landscape design of urban forest parks based on the Five Senses Theory: A case study of Stanley Park in Canada. Int J Geoheritage Parks, 10:97-112.

[41] Beard JR (2024). Beyond integrated care for older adults. Nat Aging, 4:1-4. 10.1038/s43587-023-00542-7 38177328

[42] Hamlin MJ, Yule E, Elliot CA, Stoner L, Kathiravel Y (2016). Long-term effectiveness of the New Zealand Green Prescription primary health care exercise initiative. Public Health, 140:102-108. 10.1016/j.puhe.2016.07.014 27569778

[43] Adewuyi FA, Knobel P, Gogna P, Dadvand P (2023). Health effects of green prescription: A systematic review of randomized controlled trials. Environ Res, 236:116844. 10.1016/j.envres.2023.116844 37574099

[44] Nguyen P-Y, Astell-Burt T, Rahimi-Ardabili H, Feng X (2023). Effect of nature prescriptions on cardiometabolic and mental health, and physical activity: a systematic review. Lancet Planet Health, 7:e313-e328. 10.1016/S2542-5196(23)00025-6 37019572

[45] Xi D et al. (2024). Risk factors associated with heatwave mortality in Chinese adults over 65 years. Nat Med, 30:1489-1498. 10.1038/s41591-024-02880-4 38528168

[46] Szewczenko A et al. (2023). Urban Therapy—Urban Health Path as an Innovative Urban Function to Strengthen the Psycho-Physical Condition of the Elderly. Int J Environ Res Public Health, 20:6081. 10.3390/ijerph20126081 PMC10297887 37372668

[47] Ho A (2020). Are we ready for artificial intelligence health monitoring in elder care? BMC Geriatr, 20:358. 10.1186/s12877-020-01764-9 PMC7504871 32957946

[48] Subramanian M et al. (2020). Precision medicine in the era of artificial intelligence: implications in chronic disease management. J Transl Med, 18:472. 10.1186/s12967-020-02658-5 PMC7725219 33298113

[49] Sayed N et al. (2021). An inflammatory aging clock (iAge) based on deep learning tracks multimorbidity, immunosenescence, frailty and cardiovascular aging. Nat Aging, 1:598-615. 10.1038/s43587-021-00082-y PMC8654267 34888528

[50] Zhang C, Liu S, Chen G, Harper S (2024). Green finance and the silver economy: catalyzing China’s low-carbon development. Front Environ Sci, Opinion 12.

[51] Zhang Q, Zhang H, Xu H (2021). Health tourism destinations as therapeutic landscapes: Understanding the health perceptions of senior seasonal migrants. Soc Sci Med, 279:113951. 10.1016/j.socscimed.2021.113951 33971446

